# Corticosteroid-Resistant Secondary Immune Thrombocytopenia in Smoldering Multiple Myeloma Successfully Treated With Eltrombopag: A Case Report

**DOI:** 10.7759/cureus.104525

**Published:** 2026-03-02

**Authors:** Ryutaro Tominaga, Hiroyuki Kobayashi

**Affiliations:** 1 Division of Hematology, Nasu Red Cross Hospital, Otawara, JPN

**Keywords:** eltrombopag olamine, immune mediated thrombocytopenic purpura (itp), smoldering multiple myeloma, systemic glucocorticoids, thrombocytopenia

## Abstract

Immune thrombocytopenia (ITP) is an uncommon yet clinically significant cause of thrombocytopenia in multiple myeloma (MM). We report a case of an 80-year-old female with severe thrombocytopenia identified during routine follow-up after breast cancer surgery, leading to the simultaneous diagnosis of Smoldering multiple myeloma (sMM) and secondary ITP. Laboratory findings, including an elevated immature platelet fraction, increased platelet-associated immunoglobulin G (IgG), and preserved megakaryopoiesis, supported a diagnosis of immune-mediated platelet destruction. The patient demonstrated only a transient response to corticosteroids but achieved sustained remission with eltrombopag (EPAG), while maintaining stable M-protein levels and showing no myeloma-defining events (MDEs). This report highlights the importance of considering secondary ITP in the evaluation of patients with MM.

## Introduction

Thrombocytopenia is a common and clinically significant finding in patients with multiple myeloma (MM). Its etiology is heterogeneous, including disease progression with bone marrow infiltration, treatment-related cytopenias, and infection. In addition, immune dysregulation in plasma cell dyscrasias has been associated with a broad spectrum of autoimmune manifestations [1]. Immune thrombocytopenia (ITP) is an acquired autoimmune disorder characterized by isolated thrombocytopenia resulting from accelerated platelet destruction and impaired platelet production, and it remains a diagnosis of exclusion; therefore, the evaluation must rule out other potential causes of thrombocytopenia [2].

The annual incidence of ITP is approximately two cases per 100,000 adults [3]. Although connective tissue diseases and lymphoproliferative disorders are well-established causes of secondary ITP [2], ITP may also occur in the setting of MM [1]. It has been proposed that cases associated with underlying diseases, such as systemic lupus erythematosus or HIV infection, or medications, be classified as secondary ITP [4]. The association between MM and secondary ITP is rare, often underrecognized, and optimal management strategies remain poorly defined [1]. Because thrombocytopenia in MM is often attributed to marrow involvement or treatment effects, secondary ITP may be underrecognized, potentially delaying appropriate therapy and increasing the risk of bleeding.

Smoldering multiple myeloma (sMM) is an asymptomatic precursor stage of MM, defined by clonal plasma cell proliferation and/or monoclonal protein in the absence of myeloma-defining events or end-organ damage [5]. Because optimal management of MM/sMM-associated ITP, particularly in corticosteroid-refractory cases, has not been established, reporting clinical experience remains valuable. We report a case of secondary ITP complicating sMM. This report illustrates the diagnostic challenge of distinguishing secondary ITP from other etiologies of thrombocytopenia in MM and highlights the importance of individualized treatment approaches, including the use of corticosteroids and thrombopoietin receptor agonists (TPO-RAs).

## Case presentation

An 80-year-old female undergoing routine follow-up after breast cancer surgery was referred for newly identified severe thrombocytopenia (4,000/µL). She reported no recent infections, medication changes, or systemic symptoms. Physical examination revealed scattered subcutaneous hemorrhages without lymphadenopathy or hepatosplenomegaly.

Initial laboratory evaluation revealed a normal leukocyte count, hemoglobin level, and coagulation profile. The immature platelet fraction (IPF) was elevated (13.6%), and platelet-associated immunoglobulin G (PAIgG) was markedly increased (913 ng/10⁷ cells). Serum protein analysis revealed an elevated IgG level (2,360 mg/dL) with preserved IgA and IgM levels. Immunofixation electrophoresis showed IgG-κ M protein. Serologic testing for autoimmune disorders, including antinuclear antibody, SS-A antibody, rheumatoid factor, proteinase 3-specific antineutrophil cytoplasmic antibody (ANCA), and myeloperoxidase-ANCA, was negative. Testing for Helicobacter pylori antibodies was also negative (Table 1).

**Table 1:** Laboratory data on admission WBC: white blood cell; RBC: red blood cell; PT-INR: prothrombin time-international normalized ratio; APTT: activated partial thromboplastin time; FDP: fibrin/fibrinogen degradation products; AT: antithrombin; IgG: immunoglobulin G; IgA: immunoglobulin A; IgM: immunoglobulin M; PAIgG: platelet-associated IgG; RF: rheumatoid factor; MPO-ANCA: myeloperoxidase antineutrophil cytoplasmic antibody; PR3-ANCA: proteinase3 antineutrophil cytoplasmic antibody; SS: Sjogren syndrome; CRP: C-reactive protein; BUN: blood urea nitrogen; AST: aspartate transaminase; ALT: alanine transaminase; LDH: lactate dehydrogenase; ALP: alkaline phosphatase; γGTP: γ-glutamyl transpeptidase; WT1mRNA: Wilms tumor 1 messenger ribonucleic acid

Variable	Patient value	reference range	Variable	Patient value	reference range
WBC (/µL)	4,100	3,300-8,600	Total protein (g/dL)	8.2	6.6-8.1
Neutrophil (%)	61	40-70	Albumin (g/dL)	3.6	4.1-5.1
Lymphocyte (%)	26	20-50	CRP (mg/dL)	0.06	0.00-0.14
Monocyte (%)	8	2-9	BUN (mg/dL)	13.6	8-20
Eosinophil (%)	5	1-6	Creatinine (mg/dL)	0.53	0.46-0.79
RBC (×10^6^/µL)	3.42	3.86-4.92	Sodium (mmol/L)	141	138-145
Hemoglobin (g/dL)	11.1	11.6-14.8	Potassium (mmol/L)	4.3	3.6-4.8
Platelet (×10^4^/µL)	0.4	15.8-34.8	Chlorine (mmol/L)	103	101-108
Immature platelet fraction (%)	14.6	1.1-6.1	Total bilirubin (mg/dL)	0.5	0.4-1.5
			AST (U/L)	25	13-30
PT-INR	1.08	0.85-1.15	ALT (U/L)	21	7-23
APTT (second)	27.2	22.0-33.0	LDH (U/L)	240	124-222
Fibrinogen (mg/dL)	399.4	160-350	γGTP (U/L)	15	9-32
D-dimer (µg/mL)	2.7	0-1.0	ALP (U/L)	72	38-113
FDP (µg/mL)	5	0-5	Creatinine kinase (U/L)	32	41-153
AT (%)	87	80-120	Ferittin (ng/mL)	252.3	4.63-204
			Vitamin B12 (pg/mL)	422	180-914
IgG (mg/dL)	2,360	861-1,747	Folate (ng/mL)	12.4	>4.0
IgA (mg/dL)	259	93-393	β2-microglobulin (mg/dL)	3.1	1.0-1.9
IgM (mg/dL)	35	50-269	WT1mRNA (copies/µL)	<50	<50
Free light chain-κ (mg/L)	539	3.3-19.4			
Free light chain-λ (mg/L)	25.1	5.7-26.3			
Immunofixation electrophoresis	IgG-κ M protein	negative			
PA-IgG (ng/10^7 ^cells)	913	9-25			
IgG-RF index	<2.0	<2.0			
Anti-nuclear antibody	<1:40	<1:40			
Anti-Helicobacter pylori antibody (U/mL)	7	<10			
MPO-ANCA (U/mL)	<1.0	<3.5			
PR3-ANCA (U/mL)	<1.0	<3.5			
Anti-SS-A antibody (U/mL)	6	<10			

**Figure 1:**
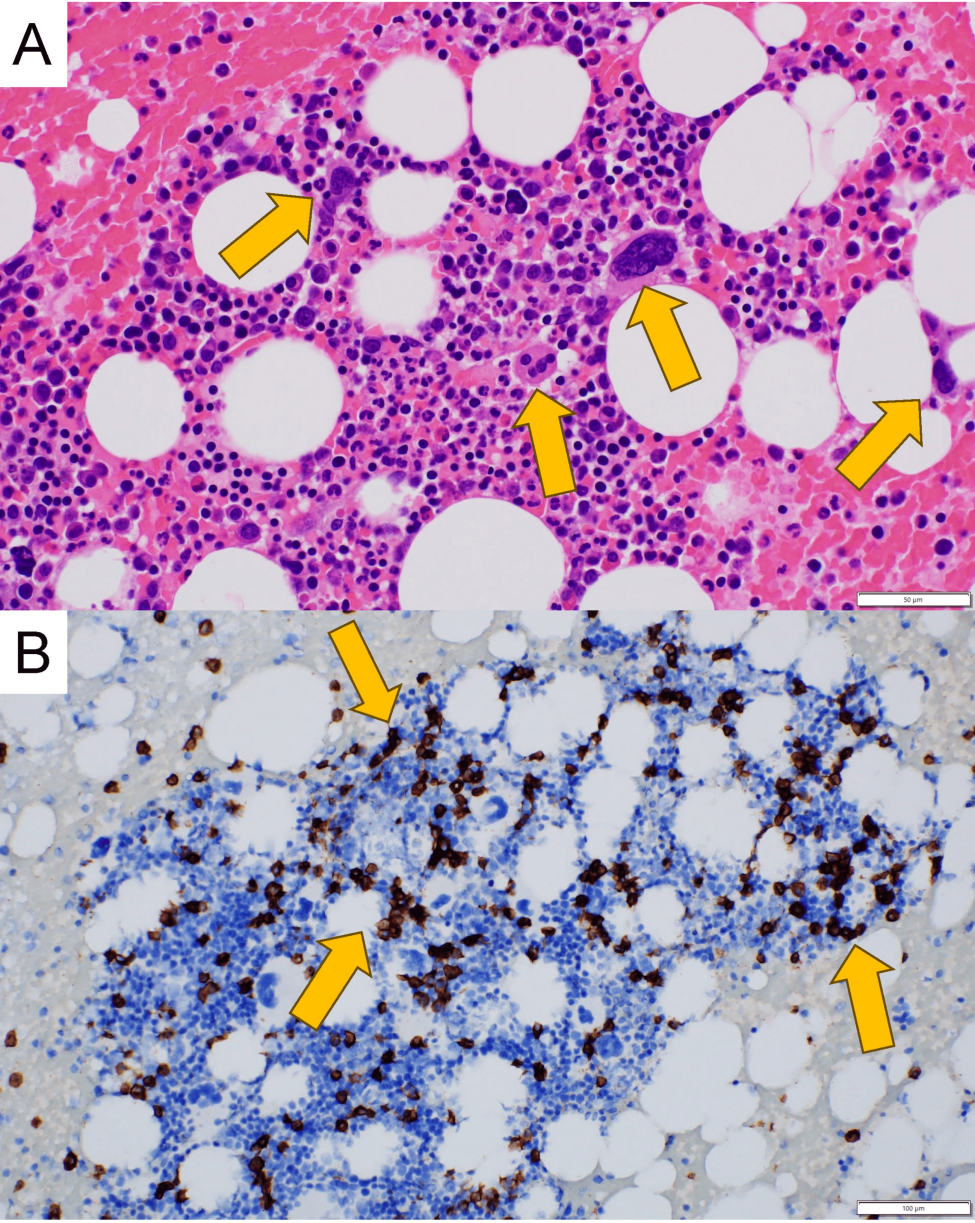
Photomicrographs of bone marrow aspirate (A) Numerous megakaryocytes in bone marrow; yellow arrows (H&E stain, ×400; scale bar = 50 μm). (B) CD138-positive monoclonal plasma cell proliferation; yellow arrows (immunohistochemistry, ×200; scale bar = 100 μm)

Given the patient’s advanced age and profound thrombocytopenia, a bone marrow examination was performed to evaluate for marrow failure, hematologic malignancy, and immune-mediated thrombocytopenia. Bone marrow examination revealed increased megakaryocytes without dysplasia and 18% plasma cells (Figure 1). Flow cytometric analysis revealed a plasma cell population with marked κ light chain restriction (κ≫λ), accounting for 21% of cells within the CD38-gated population. Imaging studies, including contrast-enhanced whole-body CT and brain MRI, revealed no evidence of infection, lytic bone lesions, or extramedullary disease. Fluorescence in situ hybridization analysis identified no high-risk cytogenetic abnormalities. These findings were consistent with sMM. Importantly, the preserved megakaryocyte compartment together with elevated IPF and PAIgG levels supported immune-mediated platelet destruction as the primary cause of thrombocytopenia.

The patient was admitted and initially treated with platelet transfusions; however, her platelet count declined further to 2,000/µL by day two. High-dose dexamethasone (20 mg/day for four days) was initiated, resulting in a transient increase in platelet count to 180,000/µL by day five [6]. Treatment was discontinued due to steroid-induced delirium, after which the platelet count decreased to 47,000/µL by day nine. Prednisolone (0.5 mg/kg/day) was subsequently initiated, but platelet counts again declined to 15,000/µL by day 17, accompanied by recurrent mucocutaneous bleeding.

**Figure 2:**
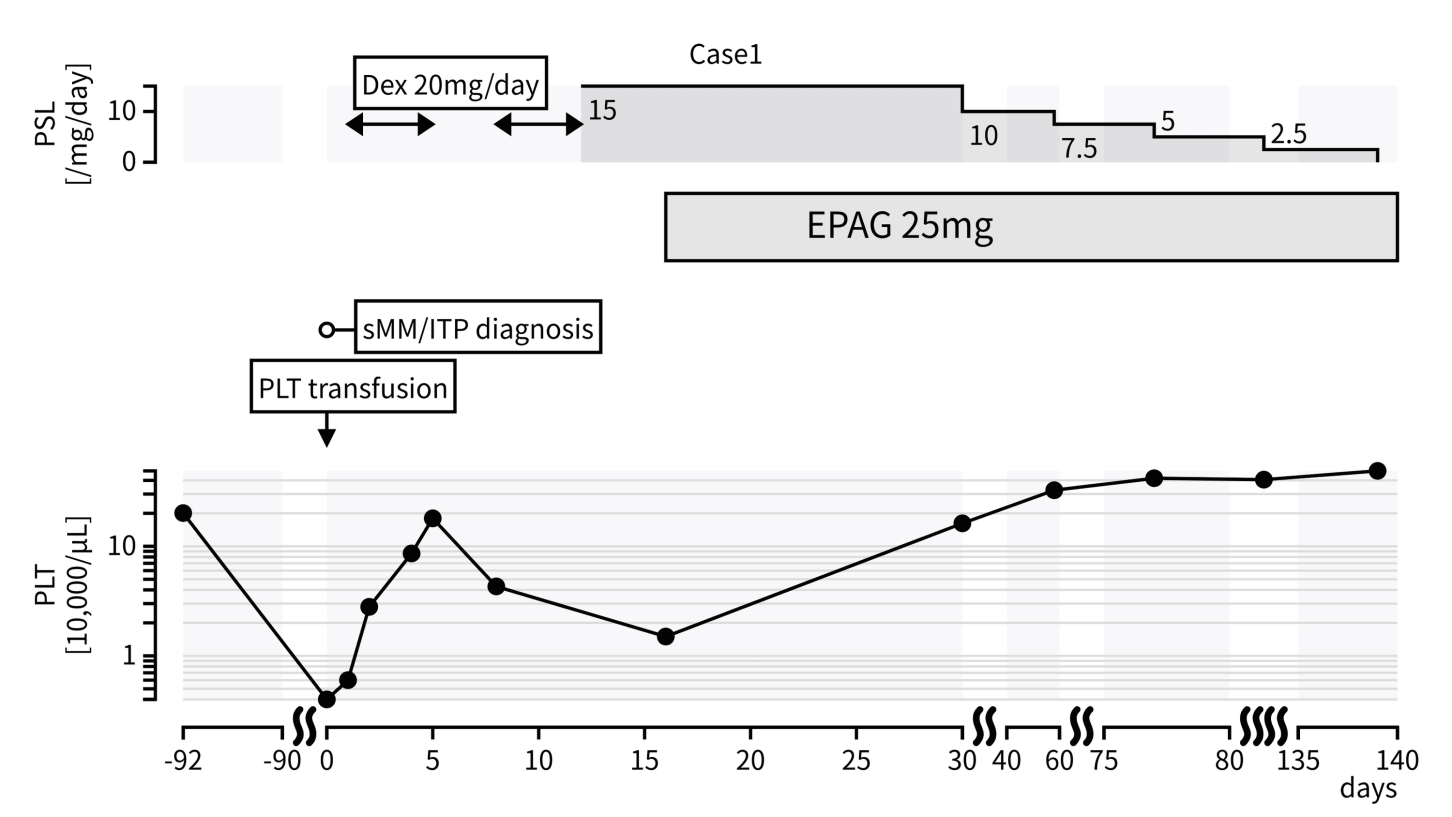
Clinical course of the case The platelet response to high-dose dexamethasone was transient. After the recurrence of thrombocytopenia, EPAG was initiated, leading to stabilization EPAG: eltrombopag

Given the inadequate and unstable response to corticosteroid therapy, we considered the possibility of steroid-refractory secondary ITP, and eltrombopag (EPAG) (25 mg/day) was initiated as second-line treatment. A gradual and sustained platelet response was observed, with counts increasing to 182,000/µL by day 31. Prednisolone was successfully tapered and discontinued over the following three months without recurrence of thrombocytopenia (Figure 2). During an additional six months of follow-up, serum IgG levels remained stable, and no myeloma-defining events (MDEs) were observed.

## Discussion

This case report describes a patient who underwent evaluation for the acute onset of severe thrombocytopenia, ultimately leading to the concurrent diagnosis of secondary ITP and sMM. Although thrombocytopenia is common in MM, the occurrence of secondary ITP in association with MM is relatively rare and must be distinguished from drug-induced cytopenias, infections, and bone marrow failure due to myeloma progression.

In our patient, thrombocytopenia progressed rapidly, whereas other hematopoietic lineages remained preserved. An elevated IPF, increased PAIgG, and megakaryocyte hyperplasia suggested that immune-mediated peripheral platelet destruction was the predominant mechanism. Although bone marrow examination revealed 18% monoclonal plasma cell infiltration, no MDEs were identified. The subsequent stability of serum IgG levels and the absence of new MDEs suggest that MM was unlikely to be the primary driver of thrombocytopenia. Although specific platelet autoantibody testing (e.g., anti-glycoprotein IIb/IIIa antibodies) was not performed, the overall laboratory and bone marrow findings were consistent with ITP, and treatment was initiated accordingly. EPAG induced a robust and sustained platelet response following a brief course of corticosteroids.

The pathophysiology of ITP is multifactorial and includes antibody-mediated platelet destruction, impaired megakaryopoiesis, T-cell dysregulation, and complement activation [6]. In MM, additional mechanisms have been proposed: monoclonal proteins may nonspecifically bind to platelets and enhance reticuloendothelial clearance, whereas myeloma-associated immune dysregulation may promote autoantibody production [1,6]. The coexistence of ITP with monoclonal gammopathy of undetermined significance and lymphoproliferative disorders is well recognized, and the present case may fall within this broader spectrum. Nevertheless, mechanistic conclusions cannot be drawn from a single case, and the association between sMM and ITP in this patient remains circumstantial.

Among previously reported cases of concurrent MM and secondary ITP [7-13], many required second-line treatment, and steroid resistance appeared more common in patients with a higher tumor burden. Although MM in our patient was untreated, EPAG led to rapid and durable platelet recovery. TPO-RAs have demonstrated efficacy in both primary ITP and secondary ITP associated with lymphoid malignancies [14]; however, reports specifically involving MM remain limited. In addition, several agents that are used to treat MM, such as bortezomib and lenalidomide, have been associated with treatment-related thrombocytopenia [15-20]. This case, therefore, provides additional clinical insights, suggesting that TPO-RAs may represent a feasible therapeutic option in selected patients with MM-associated secondary ITP.

Clinically, this report underscores the importance of considering secondary ITP early in the differential diagnosis of profound thrombocytopenia during MM evaluation, alongside marrow infiltration and treatment-related cytopenias. Furthermore, the favorable response to EPAG suggests that TPO-RAs may be effective even in untreated MM when the corticosteroid response is inadequate.

## Conclusions

Thrombocytopenia in MM arises from diverse etiologies, and secondary ITP should be recognized as a possible, treatable cause. This report illustrates that secondary ITP may develop even in the setting of stable MM disease activity and supports considering TPO-RAs as part of the therapeutic armamentarium. Continued accumulation of similar cases will be essential to clarify the pathophysiology of MM-associated secondary ITP and to optimize management strategies.
